# Tailor swiftly: lessons learned from a nationwide implementation of an antimicrobial stewardship program for asymptomatic bacteriuria

**DOI:** 10.1017/ash.2025.63

**Published:** 2025-05-26

**Authors:** Trenton M. Haltom, Sophia Braund, Rogelio Hernandez, Larissa Grigoryan, Barbara W. Trautner, Eva Amenta

**Affiliations:** 1 Center for Innovations in Quality, Effectiveness and Safety (IQuESt), Michael E. DeBakey VA Medical Center, Houston, TX, USA; 2 Department of Medicine, Section of Health Services Research, Baylor College of Medicine, Houston, TX, USA; 3 Tilman J. Fertitta Family College of Medicine, University of Houston, Houston, TX, USA; 4 Department of Family and Community Medicine, Baylor College of Medicine, Houston, TX, USA; 5 Department of Medicine, Section of Infectious Diseases, Baylor College of Medicine, Houston TX, USA

## Abstract

**Objective:**

Overtreatment of asymptomatic bacteriuria (ASB) is a major cause of antibiotic overuse. We facilitated a nationwide implementation of an ASB antimicrobial stewardship intervention in 41 Veterans Affairs facilities. Twenty-one sites participated in a Virtual Learning Collaborative (VLC) with monthly webinars. We assess what VLC teams learned from one another in these webinars.

**Methods:**

The bi-monthly VLC webinars featured expert presentations and spotlighted 1–2 site teams, asking them to discuss their barriers and facilitators for the intervention. Data come from analyses of descriptive field notes from the webinars and chat transcripts. Field notes were analyzed using the “sort and sift, think and shift” method. We sorted and labeled common strategies thematically, sifted through illustrative quotes, and iteratively discussed the results to reach consensus.

**Results:**

Across 22 webinars (August 2023–April 2024), sites discussed different resources, team membership, and organizational structures. Sites had to “tailor swiftly” to their site needs and target audiences by adapting educational materials for timing, length, audience, and outreach location. Sites used five tailoring strategies to implement the antimicrobial stewardship program: Organizational and Structural Strategies, Recruitment Strategies, Data- and Information-Based Strategies, Interpersonal Strategies, and Resource Provision.

**Conclusion:**

VLC webinars allowed sites to share tips and strategies for the implementation of a nationwide antimicrobial stewardship program wherein rapid tailoring and local adaptations were effective. Our supportive approach to tailoring allowed implementation sites to adapt antimicrobial stewardship materials and intervention delivery to their different resources and organizational contexts.

## Introduction

Asymptomatic bacteriuria (ASB) is frequently confused with urinary tract infection (UTI) in acute and long-term care settings and treated inappropriately with antibiotics.^1^ The practice of treating ASB goes against *The Infectious Diseases Society of America*and the *US Preventive Services Task Force* recommedations.^2,3^ Despite these recommendations, inappropriate treatment of ASB remains widespread. Vaughn et al. found that 43.5% of inappropriate antibiotic treatments assessed at hospital discharge were due to the treatment of ASB.^4^ Suspected UTI is one of the main reasons for prescribing antibiotics in long-term care facilities, and many of these treated “UTIs” are actually inappropriately treated asymptomatic bacteriuria.^5^

Prior small-scale antimicrobial stewardship interventions at Veterans Affairs (VA) medical centers have been effective at reducing the inappropriate treatment of ASB. One such intervention, “Kicking Catheter-Associated Urinary Tract Infection (CAUTI),” used audit and feedback approaches to achieve this goal in both acute and long-term care.^6^ The Kicking CAUTI implementation was further successful when scaled up to four geographically diverse VA facilities, wherein external facilitation was effective at reducing urine culture rates and ASB treatment, including days and length of therapy.^7^ Increased engagement with the intervention correlated with a decrease in antibiotic use among participating sites.^8^ Engagement with the intervention was important to its effectiveness, yet difficult to achieve among overworked antimicrobial stewardship teams.^9^

In the current study, we draw on data from Kicking CAUTI 2.0, a nationwide Type 3 cluster (hybrid effectiveness and implementation) randomized control trial. Kicking CAUTI 2.0 builds on former iterations of the implementation by assessing the comparative effectiveness of a Virtual Learning Collaborative (VLC) to a Technical Assistance strategy, both commonly used techniques in antimicrobial stewardship research. As the Technical Assistance sites were not invited to the webinars (as group learning experiences), we only analyze data from interactions from the VLC webinars here. Our VLC webinars provided participants with opportunities to learn from each other in a large collaborative environment.^10–12^

Extant literature has explored the impact of learning collaboratives on quality improvement initiatives, often compared to other approaches, with mixed results.^13–15^ VLCs, however, had been shown to reduce implementation costs, provide live input, and allow participants to learn from one another.^15,16^ More specifically, VLCs have been shown to be effective in achieving quality improvement in antimicrobial stewardship interventions in both acute and long-term care facilities.^17,18^

Yet, *how* VLCs encourage antimicrobial stewardship and *what* strategies emerge remains under reported.^19^ Thus, we assessed VLC participants’ strategies, lessons learned, and shared wisdom for implementing antimicrobial stewardship through qualitative analyses of VLC transcripts and chat responses that could apply across sites. We find that the ideas garnered from sharing antimicrobial stewardship strategies in VLC webinars lead participants to tailor the intervention locally and swiftly.

## Methods

**Figure 1.**
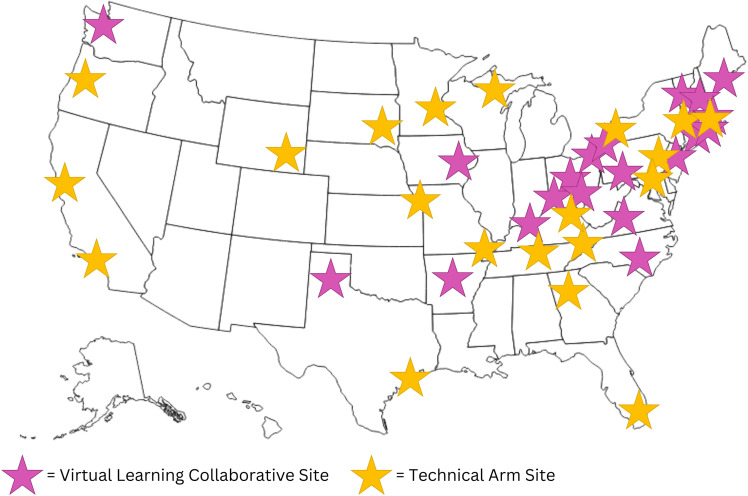
Nationwide map of participating Kicking CAUTI 2.0 VA sites. *Note:* The current study focuses only on Virtual Learning Collaborative sites.

Data come from bimonthly webinars hosted as part of Kicking CAUTI 2.0, which randomized 41 VA sites into one of two implementation strategies: a VLC or Technical Assistance. Sites could choose where to implement the intervention, but most focused on acute and long-term care units. We invited only VLC sites to participate in monthly webinars from which we derive the results presented here. See Figure 1 for a map of VA sites and Table 1 for details of webinar attendance, webinar evaluation, and VLC facility characteristics. Site teams had access to a catalog of educational materials and were to deliver teaching cases from this catalog in local clinical settings. VLC sites were encouraged (though not required) to attend learning community webinars, which offered the same content twice monthly to accommodate clinical schedules. Webinars began with an overview and update of the project, then a subject matter expert spoke for 20–25 minutes on a relevant topic. We encouraged webinar participants to use the chat function and facilitated a ten-minute Q&A session following presentations. The remaining time was spent on 1-2 “site spotlights” during which we asked team members to address the following questions: What’s working well? How have your intervention delivery sessions gone? What has been your impression of the audience’s reception? What challenges have you found?

**Table 1.** Virtual Learning Collaborative (VLC) webinar and site demographics

	*n*
*Webinars*	22
*Total Webinar Attendees*	704
Attendees per Webinar *(Avg.)*	32
*# of Sites Participating per Webinar (Avg.)*	13
*Preference to Continue Webinars Post-Intervention* (*n* = 41)*	*n* (%)
Yes	41 (100)
No	0 (0)
*Shared Webinar Information Locally* (*n* = 39)*	
Yes	32 (82)
No	7 (18)
VA Facility Complexity** (N = 21)	
High Complexity	15 (71)
Medium Complexity	1 (5)
Low Complexity	5 (24)

*Based on an informal poll fielded on Microsoft Teams to VLC participants (N = 95) during the February 13 (*n* = 43) and 29 (*n* = 52), 2024 webinars. Preference to continue webinars question total response rate: 43%. Shared webinar information question total response rate: 41%.

**There are three VA facility complexity categories (high, medium, and low complexity) which involve volume, patient risk, teaching, research capabilities, number and breadth of specialists, and number of Intensive Care Units (ICUs). There are three high-complexity levels (1a-1c); we merged them for reporting.

Data for this study come from descriptive field notes and summaries produced from each webinar by a qualitative methodologist (TMH).^20^ Webinar sessions were recorded and transcribed using MS Teams. We also saved all chat messages. Intervention team members (TMH, EA) iteratively reviewed recordings, transcripts, and chat records by individual webinar. We revised the field notes accordingly to capture the breadth and depth of antimicrobial stewardship strategies shared by guest presenters or from the site spotlights. We used the “sort and sift, think and shift” analytical approach by letting the data guide the creation of categories and patterns across webinars.^21^ We sorted and labeled common strategies thematically, sifted through illustrative quotes, and iteratively discussed the results to reach consensus. We refer to all webinar attendees as “participants.”

## Findings

We hosted 22 webinars between August 2023 and April 2024, consisting of 12 expert presentations related to diagnosis of UTI versus ASB and appropriate antibiotic use. Each webinar was attended by an average of 32 participants (*n* = 704) representing an average of 13 of the 21 sites assigned to the VLC arm. Seventy-seven percent of sites had at least one local site team member who attended one or more of the two monthly webinars. At the end of the intervention year (April 2024), we polled webinar participants about whether they would like the webinars to continue in the sustainability year. All respondents wanted to continue the webinars (*n* = 41), and most had shared the webinar information locally (*n* = 32, 82%). Most of the 21 VLC sites are considered highly complex (*n* = 15; 71%).

**Figure 2.**
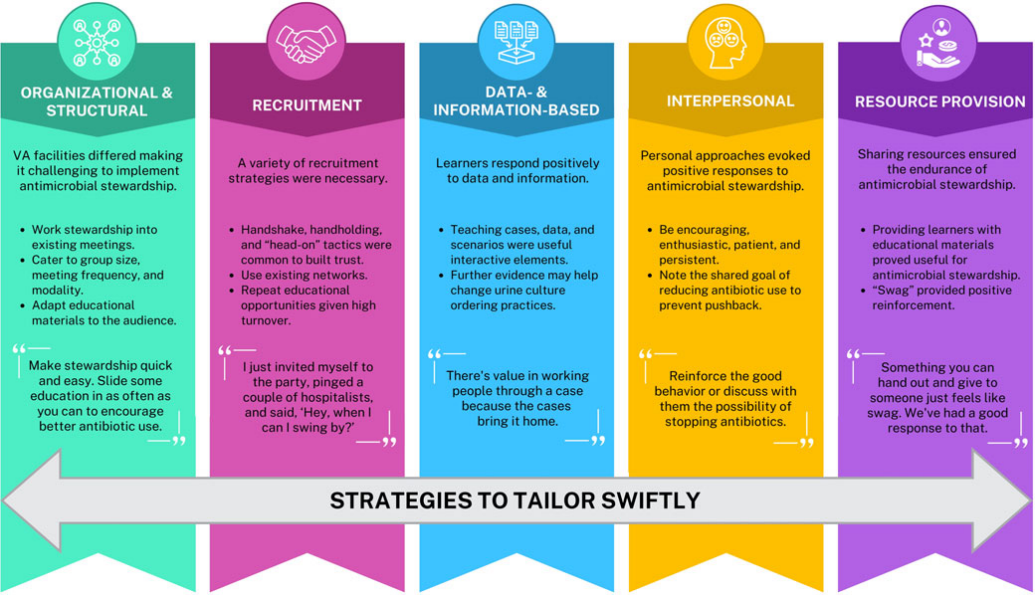
Summary of results and illustrative quotes.

As local site team members shared their antimicrobial stewardship strategies across webinars, it became clear that each implementation site had different resources, team membership, and organizational structures. For example, some sites were teaching sites with residents who rotated monthly, while at others, the target audiences for the stewardship messaging were long-term VA staff physicians and nurses. Some local teams were as small as one person (usually a pharmacist), while larger teams included an infectious diseases physician. The spotlighted teams shared their creative approaches to overcoming local challenges to implementation. Thus, teams learned from each other and adapted their antimicrobial stewardship strategies, allowing them “tailor swiftly” for effective reach and implementation. Tailoring meant adapting the delivery of the intervention (eg, antimicrobial stewardship educational materials) for timing, length, audience, and location of outreach. Teams specifically tailored using five strategies to implement the antimicrobial stewardship program: Organizational and Structural Strategies, Recruitment Strategies, Data- and Information-Based Strategies, Interpersonal Strategies, and Resource Provision (Figure 2).

### Organizational and structural strategies

Each VA facility was organized and structured differently. As implementation teams sought to conduct antimicrobial stewardship, they found it challenging to find opportunities to speak to different providers or staff groups, given everyone’s busy schedules and disparate ASB knowledge base. Despite these challenges, participants found unique ways to engage learners.

Participants worked antimicrobial stewardship into existing structures (eg, regularly scheduled presentations or meetings) to provide a variety of touchpoints and accommodate demands on learners’ time. This meant acknowledging specific audiences, needs, allotted time, and presentation modalities (ie, in-person vs. virtual) to cater to group size, meeting frequency, and convenience. Participants would present cases to clinicians during their regular team rounds, morning huddles, during grand rounds presentations to larger audiences, and even one-on-one. Teaching moments could happen at any time and in any setting. Infiltrating standing meetings allowed participants to establish a consistent pattern and relationship such that clinical teams would anticipate regular antimicrobial stewardship presentations—“they all know that we’re coming on every other Friday.”

Though participants recommended a variety of modalities, prioritizing in-person engagement seemed to avoid the challenges of virtual interactions (eg, poor engagement, few cameras on). Some participants planned special events for Continuing Medical Education (CME) credit, targeted hard-to-reach teams (eg, emergency department or night shift), or monthly nursing teaching seminars. During these exchanges, participants recommended making “stewardship quick and easy—slide some education in as often as you can to encourage better antibiotic use.” On the other hand, Microsoft Teams also “helped the flow of communication for informal questions” to facilitate this “quick and easy” approach virtually due to its wide availability throughout VA facilities and convenient chat function. In consideration of the convenience of antimicrobial stewardship, the timeliness of outreach and accommodating schedules were key.

To aid the efficiency and focus of antimicrobial stewardship, participants recommended adapting educational materials to the target audience because “different audiences need different cases—know your audience.” Another participant described how she would prioritize looking for “cases that would apply to the situation/team we will be presenting to.” Adapting materials made them more relevant to local cases or clinical specialties, thus increasing potential uptake and acceptance. Both webinar experts and webinar participants made slide decks available and would tailor them for the intended audience or depending on the length of outreach opportunities. For example, after an initial presentation, learners indicated wanting more data-based examples, so the local antimicrobial stewardship team provided more data in their second meeting. Participants also regularly invited residents and fellows to review and present cases to engage them in their own learning. One participant shared how they “frequently mix the teaching cases [provided by the Kicking CAUTI 2.0 team] with our own cases of ASB” to keep educational materials relevant to local patient populations. Antimicrobial stewardship audiences varied greatly, and participants took into consideration specialties (eg, spinal cord injury, psychiatry, acute care, home-based primary care) and locations (eg, community living centers or nursing homes) to adapt their materials accordingly.

### Recruitment strategies

To maximize antimicrobial stewardship opportunities, site teams used a variety of recruitment strategies. Handshake, handholding, and “head-on” tactics were common approaches to antimicrobial stewardship shown to build trust between the local stewardship champion and providers.^22,23^ Handshake stewardship refers to creating a person-to-person connection to provide advice about antibiotic use, often between a pharmacist and a physician. One participant explained how “I just invited myself to the party, pinged a couple of hospitalists, and said, ‘Hey, when I can I swing by?’” Another participant explained how she would reach out to clinical teams early in the week for scheduling and then again later in the week to check in about more impromptu education opportunities. This “just do it,” proactive approach (rather than waiting for an invitation) opened antimicrobial stewardship opportunities and put team members’ networks to use.

Taking advantage of one’s network by starting with close colleagues and expanding thereafter was key to recruiting multiple audiences—even temporary ones like residents on rotation. Using professional networks also helped recruit providers or nurses to become antimicrobial stewardship champions in their sections. One participant lamented the challenge of getting clinicians on board with antimicrobial stewardship without a physician champion; another participant echoed the sentiment, emphasizing the usefulness of hearing “from one of their own.” In addition, repeatedly educating new personnel at a given site was a strategy to maintain antimicrobial stewardship education and implementation effectiveness. Repeating educational opportunities were essential given the temporariness of some positions (eg, residents), high turnover, internal movement, retirement, parental leave, extended absences, etc.

### Data- and information-based strategies

Implementation teams found learners responded positively to data- and information-based approaches to antimicrobial stewardship. As part of the implementation toolkit, teams had access to a variety of interactive clinical teaching cases to present. Participants noted the “value in working people through a case because the cases bring it home.” The use of these cases, data, and scenarios was useful interactive element that would elicit responses, perspectives, and feedback to highlight the rationale behind decisions. During one check-in, participants expressed how high-level learners like residents were especially interested in seeing supporting data. Participants would also review definitions of key concepts to align knowledge and reveal discrepancies like conflicting definitions of UTI in European and United States guidelines.^24,25^ These conflicting guidelines made the implementation of antimicrobial stewardship confusing and, as a result, some providers were stuck in their ways, making it “hard to break some of those habits” (in reference to sending unnecessary urine cultures for analysis). Using data as evidence helped balance hospital-wide practices with those stubborn to change their urine culture ordering practices.

### Interpersonal strategies

Throughout webinars, both experts and participants emphasized ways to approach antimicrobial stewardship on a personal level. In general, clinicians’ responses to receiving the antimicrobial stewardship intervention were positive. One participant reported that they “get positive comments. We’ve had providers tell us that we are helping to shape changing their practice.” The positivity stems from the demeanor site teams used, such as showing encouragement and enthusiasm, and having patience and persistence. One participant described presenting one or two cases a week, which proved to be good reminders and reinforced practices. These strategies lead to opportunities to “reinforce the good behavior or discuss with them the possibility of stopping antibiotics.” Participants were cognizant of providers’ varied responses to their antimicrobial stewardship, which ranged from enthusiastic to hesitant, with suspicions of being micromanaged. To alleviate these concerns, participants would reach out to providers who frequently ordered urine cultures and noted the shared goal of reducing antibiotic use to prevent pushback. One participant shared the scenario of a specialist who expressed they did not need antimicrobial stewardship advice because “we’re experts at UTIs.” The participant responded, “That’s great! Can you help us teach?” This ego-syntonic approach with self-designated experts offers the opportunity to teach specialists and use their clout to educate others. Encouraging positive demeanors and promoting teamwork allow for successful antimicrobial stewardship.

### Resource provision

Resources and sharing of these resources were key to ensuring antimicrobial stewardship endured. Beyond interactive case scenarios, the Kicking CAUTI team provided tools for positive reinforcement using “swag,” including sticky notes with the study logo and pocket cards with a diagnostic algorithm (flow chart) for UTIs/ASB. Participants appreciated these items because “having something you can hand out and give to someone just feels like swag. We’ve had a good response to that.” In reference to the pocket cards, the same participant elaborated how “giving someone something they can hold on to and say, ‘I’m just doing what this says,’” makes the decision to order a urine culture less personal. Webinars provided participants to share their own locally developed resources, like urine culture order sets or order menus; fellow participants showed keen interest in these for their own facilities. Beyond free swag, providing learners with educational materials proved useful for antimicrobial stewardship. As members of Kicking CAUTI, all participants had access to a plethora of tools via Microsoft SharePoint, a central website used to store, organize, and share information. Participants would also adapt resources or recreate them for quick sharing.

## Discussion and conclusion

Kicking CAUTI 2.0 sites adapted antimicrobial stewardship materials to their needs by “tailoring swiftly” in ways that accounted for differing resources and organizational contexts. The VLC webinars provided opportunities for sharing these lessons across sites and teams with varying resources. Implementation teams used organizational and structural strategies to educate about antimicrobial stewardship, developed recruitment strategies, brought in evidence to support antimicrobial stewardship, built upon interpersonal relationships, and provided helpful resources. We summarize these strategies in Figure 2.

Implementation of an intervention poses challenges in how to maintain engagement to maximize its impact. For example, “trialability,”^26^ the option to try the intervention and change or stop based on local experience, is related to the tailoring teams reported in webinars. Site teams shared tips and strategies for antimicrobial stewardship in our nationwide program, wherein swift tailoring and focused strategies proved effective. Participants reported that being creative and flexible kept antimicrobial stewardship fresh and interesting for learners across implementation sites. Furthermore, providing a series of tools (eg, an implementation guide, teaching cases, swag, etc.) aided engagement with the antimicrobial stewardship intervention. The educational component of presentations from content experts during webinars supplemented the materials we provided and encouraged antimicrobial stewardship. This collection of resources allowed intervention teams to trial their approaches and make changes with the shared goal of successfully deducing inappropriate treatment with antibiotics and inappropriate treatment of asymptomatic bacteriuria (ASB) per the Consolidated Framework for Implementation Research.^27^

A VLC provides a unique benefit because it offers opportunities for participating sites to learn from each other about how to individually adjust or tailor the intervention to maximize its utility at their site.^13–15,28^ Our findings are in line with similar studies that found local tailoring to be beneficial in implementation of an intended intervention. A national implementation intervention to reduce rates of Catheter-Associated UTIs in nursing homes used monthly calls with participating sites to share barriers and successes.^18^ The intervention was successful in reducing rates of UTI diagnosis in nursing homes as a result. Likewise, an international antimicrobial stewardship intervention to reduce rates of inappropriate treatment of UTIs used a participatory action research model wherein the research team met with participating sites to review materials and help with local tailoring based on the individual country’s needs and reflection of the adaptation of the intervention.^29^ This international program found a clinically relevant reduction in inappropriate antibiotic treatment of UTIs without an increase in adverse events or all-cause mortality.^29^ Both of these examples showcase the benefits of iterative adaptation or tailoring of an intervention to achieve the intended outcome.

Overall, the VLC strategy encouraged engagement throughout the intervention by offering novel expert perspectives and implementation tips from peers. Our review of webinars hosted by a nationwide antimicrobial stewardship intervention reveals how VLC sites learned from each other, thus encouraging swift tailoring of antimicrobial stewardship efforts using ideas generated from webinar sessions. The timeliness of the webinars further aided swiftness—if a site was stuck or out of ideas, our twice-monthly webinars offered fresh perspectives. VLCs are a widely used implementation strategy in VA and non-VA settings, and the lessons learned from our VLC about tailoring swiftly may be applicable to other integrated healthcare systems, as the VA is one of the largest integrated healthcare networks in the United States.

## Data Availability

Not relevant. Data will not be made publicly available.
